# Systematic review and meta-analysis of risk scores in prediction for the clinical outcomes in patients with acute variceal bleeding

**DOI:** 10.1080/07853890.2021.1990394

**Published:** 2021-10-18

**Authors:** Ling Yang, Rui Sun, Ning Wei, Hong Chen

**Affiliations:** Department of Gastroenterology, Zhong Da Hospital, Medical School, Southeast University, Nanjing, PR China

**Keywords:** Risk score, acute variceal bleeding, prognosis, meta-analysis

## Abstract

**Background:**

Acute variceal bleeding (AVB) is a life-threatening condition that needs risk stratification to guide clinical treatment. Which risk system could reflect the prognosis more accurately remains controversial. We aimed to conduct a meta-analysis of the predictive value of GBS, AIMS65, Rockall (clinical Rockall score and full Rockall score), CTP and MELD.

**Method:**

PubMed, Web of Science, Embase, Cochrane library, WANGFANG and CNKI were searched. Twenty-eight articles were included in the study. The Meta-DiSc software and MedCalc software were used to pool the predictive accuracy.

**Results:**

Concerning in-hospital mortality, CTP, AIMS65, MELD, Full-Rockall and GBS had a pooled AUC of 0.824, 0.793, 0.788, 0.75 and 0.683, respectively. CTP had the highest sensitivity of 0.910 (95% CI: 0.864–0.944) with a specificity of 0.666 (95% CI: 0.635–0.696). AIMS65 had the highest specificity of 0.774 (95% CI: 0.749–0.798) with a sensitivity of 0.679 (95% CI: 0.617–0.736). For follow-up mortality, MELD, AIMS65, CTP, Clinical Rockall, Full-Rockall and GBS showed a pooled AUC of 0.798, 0.77, 0.746, 0.704, 0.678 and 0.618, respectively. CTP had the highest specificity (0.806, 95% CI: 0.763–0.843) with a sensitivity of 0.722 (95% CI: 0.628–0.804). GBS had the highest sensitivity 0.800 (95% CI: 0.696–0.881) with a specificity of 0.412 (95% CI: 0.368–0.457). As for rebleeding, no score performed particularly well.

**Conclusions:**

No risk scores were ideally identified by our systematic review. CTP was superior to other risk scores in identifying AVB patients at high risk of death in hospital and patients at low risk within follow-up. Guidelines have recommended the use of GBS to risk stratification of patients with upper gastrointestinal bleeding. However, if the cause of upper gastrointestinal bleeding is suspected oesophageal and gastric varices, extra care should be taken. Because in this meta-analysis, the ability of GBS was limited.

**Key message**
- CTP was superior in identifying AVB patients at high risk of death in hospital and low risk within follow-up.
- GBS, though recommended by the Guidelines, should be cautiously used when assessing AVB patients.

## Introduction

Acute variceal bleeding (AVB) is one of the leading causes of acute upper gastrointestinal bleeding (AUGIB), and the incidence is second only to peptic ulcers [1]. They are most often a consequence of portal hypertension [2], commonly due to cirrhosis. Varices can be found in 50% of cirrhotic patients, and they develop at a rate of 5–15% per year [3]. The variceal bleeding may be brisk, and patients may soon develop shock. The 6 weeks mortality with each episode of variceal haemorrhage is approximately 20% [3].

Quick and precise treatment can reduce mortality. Risk stratification could help recognize the high-risk patient, resulting in closer monitoring, faster response and improved prognosis. The consensuses proposed that risk stratification scores should be used as soon as possible in patients with AUGIB including ulcer and nonvariceal bleeding [4–6]. However, it is not until the endoscopy has been completed that the cause of bleeding would be known. As long as upper gastrointestinal bleeding is suspected, an urgent risk assessment should be done. Besides, AVB, as a much more dangerous condition than nonvariceal bleeding, is more in need of stratification scores. Up to now, several risk scores have been invented. The most widely used scores for predicting upper gastrointestinal bleeding are the Glasgow-Blatchford score (GBS), the Rockall score and the AIMS65 score. In 2000, the GBS was developed and validated to predict in-hospital rebleeding, death and the need for intervention [7]. The Rockall score was created in 1996 to predict death and rebleeding [8], including clinical Rockall score and full Rockall score. Saltzman et al. developed and validated the AIMS65 score in 2011 to predict in-hospital death [9]. However, the three risk scores were validated and compared mostly in AUGIB patients, especially acute nonvariceal upper gastrointestinal bleeding (ANVUGIB). Patients with variceal bleeding were excluded or only accounted for a small part. The best score predicting the prognosis of AVB patients remains unclear. Another two predictive scores for patients with chronic liver disease are also gradually used in predicting variceal bleeding. The Child–Pugh score (CTP) is a valuable tool for determining the prognosis of chronic liver disease, especially cirrhosis [10]. Another scoring system for assessing the seriousness of the chronic liver disease is the model for end-stage liver disease (MELD). It is commonly used to estimate mortality in patients who had a transjugular intrahepatic portosystemic shunt (TIPS) procedure [11] and prioritize for receipt of a liver transplant [12]. Previous studies had reported the two staging systems’ predictive abilities in AVB patients’ outcomes, but which could reflect the prognosis more accurately remained controversial [13,14]. We aim to conduct a systematic review of the predictive value of GBS, AIMS65, Rockall (clinical Rockall score and full Rockall score), CTP and MELD in risking stratify AVB patients for mortality and rebleeding within three months after the initial bleeding.

## Methods

### Search strategy

“Variceal bleeding” and “risk scor*” were searched in PubMed, Web of Science, Embase, the Cochrane library, WANGFANG (Chinese) and CNKI (Chinese) from inception to February 2021. (The detailed search strategy showed in Supplement materials). All search results were exported to the EndNote version 8 (Thomson Reuters, Toronto, Canada).

### Study selection

Eligible articles ought to meet the following criteria: 1) adults (≥18 years) who presented with AVB, confirmed by upper GI endoscopy (oesophageal, fundal, or both) 2) studies concerning GBS, AIMS65, Rockall (clinical Rockall score and full Rockall score), CTP or MELD score were included in this meta-analysis. 3) All risk scores should be consistent with the internationally recognized standard. The exclusion criteria included duplicate articles, reviews, letters to the editor, case reports, animal studies and children studies. With the exception of duplicates, two reviewers (L.Y. and N.W.) independently screened the titles and abstracts of all reported studies. The full texts of the selected papers were then scanned separately, and the eligibility and exclusion requirements were applied. Disagreements were addressed and settled by a discussion.

### Outcome measures

Outcomes included mortality and rebleeding. Mortality was defined as all-cause death, including in-hospital death and follow-up death within three months. Rebleeding was defined as variceal bleeding that happened again after a 24-h clinical stable period by haemostasis, which included in-hospital rebleeding and follow-up bleeding within three months. Follow-up time within seven days was considered to be in hospital.

### Data abstraction

Two reviewers (L.Y. and N.W.) extracted data from eligible articles. A third reviewer (H.C.) was consulted when facing the divergence. The following variables were collected from the included articles: author names, country, year of publication, study design, demographics and samples, the sensitivity, specificity, positive predictive value (PPV), negative predictive value (NPV), suggested cut-offs, the area under the receiver-operating characteristic curve (AUC), 95% CIs and SEs. In principle, if a test had an AUC lower than 0.5, the data were not included into meta-analysis [15].

### Quality assessment

QUADAS-2 tool was used to assess the risk of bias and quality of included articles [16]. This tool evaluates the risk of bias from four aspects: patient selection, index test, reference standard, flow and timing of the study. For this study, the index tests are the validated risk scores. The patient outcomes within follow-up are the reference standards.

### Statistical analysis

The ability of each scoring system to predict the outcomes (mortality and rebleeding) was assessed mainly by calculating the AUC. In this meta-analysis, AUCs or SEs were used. If the SEs were not reported in the studies, it was calculated as follows (SE = upper limit of 95% CI – the lower limit of 95% CI/(2*1.96)) [17]. The use of the random effects model or fixed-effects model was dependent on the heterogeneity of studies. Subgroup analysis for in-hospital and follow-up outcomes was performed in the study. Pooled AUC of 0.5 was considered to have no predictive power, > 0.5 and ≤ 0.7 was considered poor predictive power, > 0.7 and ≤ 0.9 was considered excellent predictive power and one was considered a perfect measure [18]. For the statistical analyses, MedCalc version 15.2 (Ostend, Belgium) was used. *p* < .05 was deemed significant. We also pooled the sensitivity, specificity and positive and negative likelihood ratios. Meta-DiSc version 1.4 (Ramony Cajal Hospital, Madrid, Spain) was used for the assessment of heterogeneity and calculating the *I*^2^ statistic.

## Results

### Selection of studies

**Figure 1.**
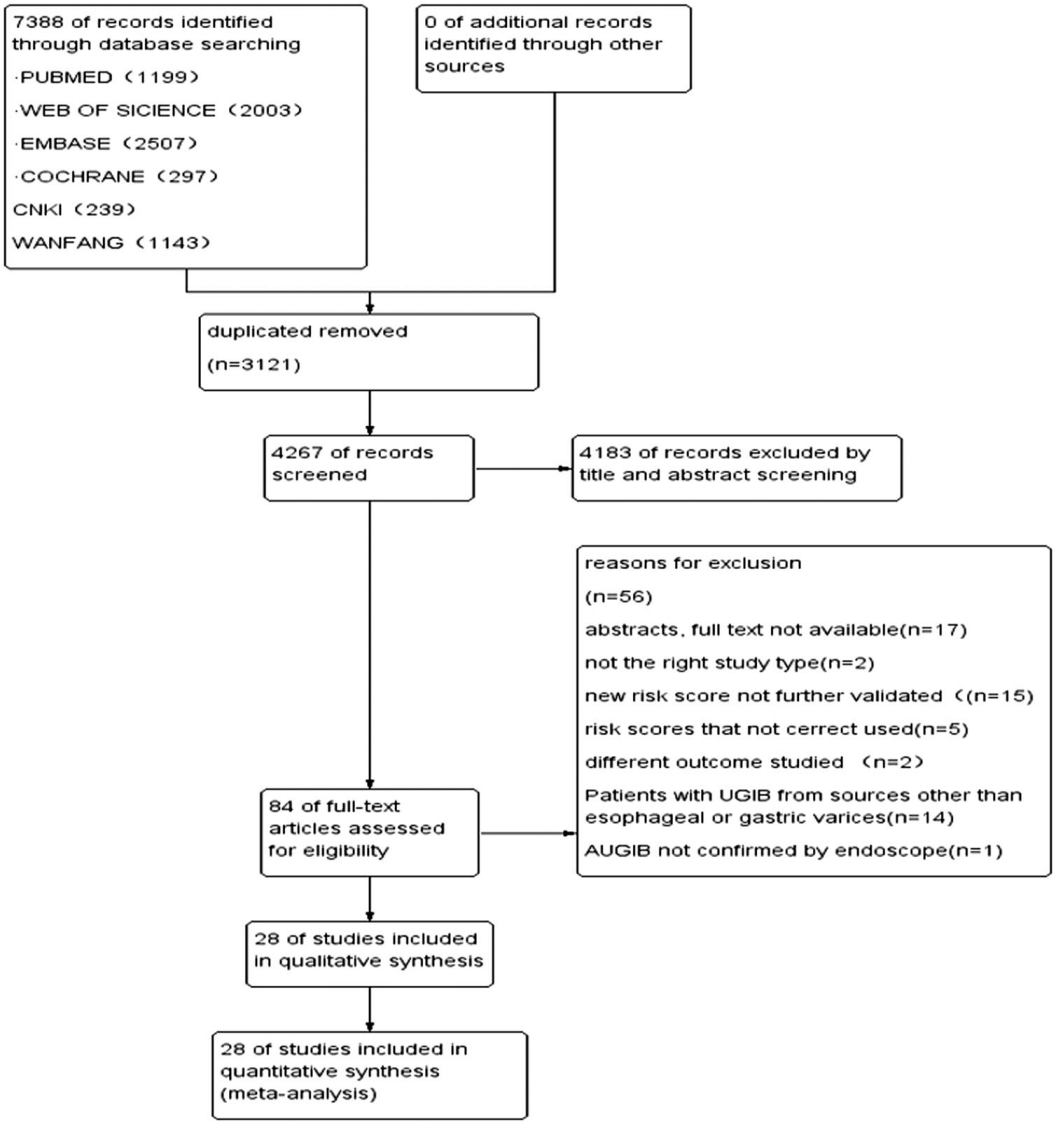
Flow chart showing the process for selecting eligible studies in this meta-analysis.

Through the electronic search, a total of 7388 articles were found. After removing the 3121 duplicates, the lefts were scanned and applied inclusion and exclusion criteria. We excluded 4183 studies after reading the titles and abstracts. Eighty-four articles were read for full-text review. Seventeen articles were excluded because the full texts were unable to obtain. Two reviews were excluded for not having the right study type. Fifteen studies studied other scores which were new or not validated and needed further research. Another five studies were excluded as the scores were not correctly calculated. Two articles were excluded as they did not measure the outcome of interest. Fourteen studies applied the predictive value of risk scores in cirrhosis patients with other reasons-caused upper gastrointestinal bleeding. And one study was excluded because the UGIB was not confirmed by an endoscope. At last, 28 articles were included in the study [13,14,19–44] (see Figure 1).

### Descriptive overview of included articles

Twenty-eight studies were included in this review (Table 1). Studies included were published between the years 2005 and 2020. All studies were conducted within a 3-month follow-up to assess mortality and rebleeding outcomes. Eleven out of 28 studies were prospective. Nine studies reported sensitivity and specificity values [19,21,26,27,32,34,36,38,42]. All studies presented AUCs and 95% CIs. An overview of the above five scores is shown in Supplementary materials.

**Table 1.** Characteristics of the included studies.

First author, study	Country	Design^a^	Number of patients	Males (%)	Age (year) (mean ± SD or range)	Risk scores	Outcomes
Zhao [14]	Australia	1	379	224 (59.1%)	53:7 ± 1:3	Child–Pugh MELD	In-hospital death
Robertson [42]	Australia	1	222	173 (78%)	56 (18–88)	AIMS65 clinical Rockall full Rockall MELD Child–Pugh	In-patient mortality, 6-week mortality and in-patient rebleeding,
Chang [41]	Thailand	2	70	55 (78.6%)	56.1 ± 12.7	AIMS65 GBS full Rockall	In-hospital death and in-hospital rebleeding^b^
Buckholz [40]	New York	–	223	156 (70%)	61 (–)	Child–Pugh MELD	6-week mortality
Tantai [38]	China	1	330	203 (61.5%)	54.9 ± 12.7	Child–Pugh MELD clinical Rockall GBS AIMS65;	In-hospital rebleeding and in-hospital mortality
Rout [37]	India	2	572	474 (82.9%)	43.5 ± 13.6	Clinical Rockall full Rockall, GBS AIMS65	42-d mortality and 42-d rebleeding
Chandnani [36]	India	2	141	40 (28.36)	–	Full Rockall, GBS AIMS65	30-d death and 30-d rebleeding
Wang [34]	China	1	202	150 (74.3%)	56.8 ± 11.8	AIMS65 GBS full Rockall MELD Child–Pugh	6-week mortality
Mandal [33]	USA	2	75	51 (67.7%)	52.5 (–)	Child–Pugh MELD	In-hospital mortality
Hassanien [32]	Egypt	1	714	500 (70%)	57.59 ± 0.46	Child–Pugh MELD AIMS65	In-hospital mortality
Iino [31]	Japan	1	47	39 (83.0%)	60 (56–67)	GBS Child–Pugh MELD	1-week mortality and 6-week mortality
Fortune [30]	USA	2	70	53 (75.7%)	51 (48–57)	Child–Pugh MELD	6-week mortality
Choe [29]	Korea	1	286	198 (69.2%)	57.9 (23–97)	GBS, full Rockall ; AIMS65	In-hospital mortality, 30-d mortality and 30-d rebleeding
Mohammad [27]	Egypt	2	120	92 (76.67%)	56.94 ± 9.20	Child–Pugh AIMS65: MELD	In-hospital mortality
Budimir [26]	Croatia	1	225	162 (72%)	61.3 ± 11.57	GBS clinical Rockall AIMS65	30-d rebleeding and 30-d mortality
Reed [25]	Scotland	2	71	43 (61%)	56 (–)	GBS full Rockall clinical Rockall	3-month mortality and 3-month rebleed
Sempere [21]	Spain	1	201	142 (70.6%)	59.48 ± 11.78	Child–Pugh MELD score	6-week mortality and 3-month mortality
Flores [13]	México	1	212	145 (68.4%)	53 ± 12	Child–Pugh MELD score	In-hospital mortality
Dunckley [20]	–	1	63	–	–	MELD, full Rockall Child–Pugh GBS	In-patient mortality and 30-d rebleed
Amitrano [19]	Italy	2	172	108 (62.79%)	61.3 ± 11.4	MELD Child–Pugh	6-week mortality and 3-month mortality
Su [43]	China	1	182	89 (48.9%)	59.7 ± 11.9	MELD GBS AIMS65	In-hospital mortality and in-hospital rebleed
Wang [22]	China	1	365	290 (79.5%)	48.8 (25–85)	MELD Child–Pugh	3-month rebleeding
Guo [44]	China	2	82	49 (59.8%)	56.74 ± 6.41	MELD AIMS65	2-month mortality and 2-month rebleeding
Gao [39]	China	1	270	105 (38.9%)	69.5 (50–86)	GBS AIMS65	In-hospital rebleeding and in-hospital mortality
Jin [35]	China	2	110	71 (64.5%)	53.5 ± 18.2	MELD, AIMS65	6-week mortality and 6-week rebleeding
Wang [28]	China	2	152	108 (71.1%)	53.56 ± 15.93	AIMS65	30-d mortality and 30-d rebleeding
Jiang [24]	China	1	101	62 (61.4%)	63.6 ± 14.8	Child–Pugh MELD	30-d mortality
Fang [23]	China	1	104	57 (54.8%)	53.2 ± 8.6	Child–Pugh MELD	3-month mortality and 3-month rebleeding

^a^ 1: retrospective; 2: prospective; “–”: not mentioned

^b^ AUCs were less than 0.5, and were not included into meta-analysis.

### Risk of bias/quality of studies

The risk of bias and applicability of the included studies was low in twenty-eight studies. Four research did not specify whether patients were enrolled consecutively, nor did they specify exclusion criteria, raising a high risk of bias in patient selection [20,22,23,44]. The results of the reference standard were not known before calculating the risk scores in any of the studies. As a result, the reference test in all research had a low risk of bias. The index test had a low risk of bias.

### Outcomes of meta-analysis

Analysis of the diagnostic threshold suggested no heterogeneity caused by the threshold effect in this study. The pooled AUC values by MedCalc version 15.2 are shown in Tables 2, 3, 5 and 6. The information was stratified by follow-up time.

**Table 2.** Predicting properties of scores for in-hospital mortality.

Scores	*N* (studies)	AUC	SE	95% CI	*p* Value
CTP	9	0.824	0.0102	0.804–0.844	<.001
AIMS65	8	0.793	0.0475	0.700– 0.886	<.001
MELD	10	0.788	0.0269	0.735–0.840	<.001
Full-Rockall	5	0.75	0.0474	0.657–0.843	<.001
GBS	7	0.683	0.0364	0.611–0.754	<.001

**Table 3.** Predicting properties of scores for follow-up mortality.

Scores	*N* (studies)	AUC	SE	95% CI	*p* Value
MELD	11	0.798	0.0134	0.772–0.824	<.001
AIMS65	9	0.77	0.0214	0.728–0.812	<.001
CTP	10	0.746	0.0358	0.675–0.816	<.001
Clinical Rockall	3	0.704	0.0292	0.647–0.761	<.001
Full-Rockall	6	0.678	0.0365	0.606–0.749	<.001
GBS	7	0.618	0.0183	0.582−0.654	<.001

**Table 5.** Predicting properties of scores for in-hospital rebleeding.

Scores	*N* (studies)	AUC	SE	95% CI	*p* Value
Clinical Rockall	2	0.689	0.0318	0.627–0.752	<.001
CTP	2	0.688	0.0307	0.627–0.748	<.001
MELD	3	0.586	0.0383	0.511–0.661	<.001
GBS	3	0.576	0.0247	0.528–0.624	<.001
AIMS65	4	0.557	0.0208	0.516–0.597	<.001

**Table 6.** Predicting properties of scores for follow-up rebleeding.

Scores	*N* (studies)	AUC	SE	95% CI	*p* Value
AIMS65	7	0.682	0.0347	0.614–0.750	<.001
CTP	2	0.661	0.0335	0.595–0.727	<.001
MELD	4	0.648	0.0533	0.544–0.753	<.001
Clinical Rockall	3	0.616	0.041	0.536–0.696	<.001
Full-Rockall	5	0.610	0.0217	0.567–0.652	<.001
GBS	6	0.578	0.0197	0.540–0.617	<.001

### Mortality

Concerning in-hospital mortality, CTP, AIMS65, MELD, Full-Rockall and GBS had a pooled AUC of 0.824, 0.793, 0.788, 0.75 and 0.683, respectively (Table 2). For follow-up mortality, MELD, AIMS65, CTP, Clinical Rockall, Full-Rockall and GBS showed a pooled AUC of 0.798, 0.77, 0.746, 0.704, 0.678 and 0.618, respectively (Table 3).

Some included studies did not involve sensitivity and specificity, and then we pooled SEN, SPE, PLR, NLR and DOR of the remaining researches by Meta-DiSc version 1.4. Concerning the total mortality, CTP had a high sensitivity (0.848, 95% CI: 0.805–0.885) and a good specificity (0.707, 95% CI: 0.682–0.731). According to follow-up time, we conducted a subgroup analysis. Regarding in-hospital mortality, CTP had a high sensitivity of 0.910 (95% CI: 0.864–0.944) and a specificity of 0.666 (95% CI: 0.635–0.696). As for follow-up mortality, GBS had the highest sensitivity of 0.800 (95% CI: 0.696–0.881) and a specificity of 0.412 (95% CI: 0.368–0.457) (Table 4). Concerning the specificity of total mortality, AIMS65 showed the highest value of 0.766 (95% CI: 0.745–0.787), with a sensitivity of 0.660 (95% CI: 0.606–0.710). In subgroup analysis according to follow-up time, AIMS65 had the highest specificity 0.774 (95% CI: 0.749–0.798) in in-hospital mortality with a sensitivity of 0.679 (95% CI: 0.617–0.736), while CTP had the highest specificity of 0.806 (95% CI：0.763–0.843) in follow-up mortality with a sensitivity of 0.722 (0.628–0.804), seeing (Table 4).

**Table 4.** Overview of overall diagnostic accuracy of the scores in mortality.

Score	Time	No studies	No. patients	Sensitivity 95% CI	Specificity 95% CI	Positive likelihood ratio 95% CI	Negative likelihood ratio 95% CI	DOR 95% CI
CTP	Total	6	1679	0.848 0.805–0.885	0.707 0.682–0.731	2.851 2.579–3.152	0.213 0.096– 0.472	14.501 6.846–30.719
	In-hospital	3	1175	0.910 0.864–0.944	0.666 0.635–0.696	2.747 2.471–3.054	0.134 0.088–0.205	20.722 12.884–33.328
	Follow-up	3	504	0.722 0.628–0.804	0.806 0.763–0.843	3.192 2.495–4.084	0.356 0.166–0.761	10.917 3.296–36.166
MELD	Total	7	1851	0.806 0.762–0.845	0.741 0.718–0.763	3.293 2.705–4.007	0.285 0.192–0.424	12.861 9.625–17.186
	In-hospital	3	1175	0.860 0.807–0.903	0.725 0.696–0.753	3.380 2.454–4.655	0.205 0.148–0.283	14.579 9.726–21.854
	Follow-up	4	676	0.724 0.644–0.795	0.768 0.730–0.804	3.349 2.381–4.711	0.354 0.272–0.461	10.723 6.976–16.481
GBS	Total	4	898	0.783 0.696–0.854	0.493 0.457–0.529	1.509 1.214–1.876	0.491 0.244–0.986	3.188 1.282–7.924
	In-hospital	1	330	0.743 0.567–0.875	0.627 0.569–0.682	1.9922 NM	0.41 NM	NM
	Follow-up	3	568	0.800 0.696–0.881	0.412 0.368–0.457	1.373 1.062–1.775	0.484 0.166–1.416	2.808 0.772–10.210
AIMS65	Total	7	1966	0.660 0.606–0.710	0.766 0.745–0.787	3.431 2.271–5.183	0.455 0.390–0.532	7.248 3.932–13.361
	In-hospital	4	1398	0.679 0.617–0.736	0.774 0.749–0.798	4.409 2.350–8.274	0.372 0.230–0.601	11.699 4.009–34.143
	Follow-up	3	568	0.600 0.484–0.708	0.748 0.707–0.786	2.501 1.375–4.549	0.540 0.411–0.709	4.425 2.758–7.099
CRS	Total	2	555	0.851 0.750–0.923	0.638 0.594–0.681	2.305 0.986–5.387	0.208 0.027–1.596	11.218 0.772–163.01
	In-hospital	1	330	0.943 0.808–0.993	0.732 0.678–0.782	NM	NM	NM
	Follow-up	1	225	0.77	0.499	NM	NM	NM
FRS	Total = follow-up	2	343	0.732 0.571–0.858	0.546 0.488–0.603	1.754 0.567–5.428	0.509 0.318–0.817	4.688 2.084–10.546

NM: not mentioned; CRS: clinical Rockall, FRS: full Rockall

### Rebleeding

As for rebleeding, no score performed exceptionally well. In predicting in-hospital recurrent bleeding, clinical Rockall had the highest predictive value of AUC (0.689, 95% CI: 0.627–0.752) (Table 5). Regarding follow-up rebleeding, AIMS65 showed the highest predictive value of AUC (0.682, 95% CI: 0.614–0.750) (Table 6). As no score had an AUC over 0.7, it showed low predictive power regardless of following-up time.

## Discussion

AVB, as a critical emergency, has the characteristics of fast bleeding, high fatality rate and high rebleeding rate. It is the most life-threatening complication of liver cirrhosis. In recent years, with the continuous development of new drugs, endoscopic intervention and other new diagnosis and treatment technologies, the mortality and rebleeding rate of AVB have declined. Despite this, the mortality at six weeks is still around 20% [3]. To provide a reference for follow-up treatment, accurately predicting the outcomes of oesophageal gastric variceal bleeding through the risk scoring system has become a research hotspot for clinicians.

This is the first meta-analysis to examine the predictive value of risk scores in AVB patients to the best of our knowledge. CTP, AIMS65 and MELD showed good predictive power for mortality in hospitals and follow-up. Full-Rockall showed good predictive power for in-hospital mortality and low predictive power for follow-up mortality. Clinical Rockall showed good predictive power in follow-up mortality. GBS has low predictive power regardless of follow-up time. As for rebleeding, no score showed good predictive power.

## CTP

CTP score and classification is uncomplicated and classical, which have long been used to evaluate liver function reserve, surgical risk and prognosis [10]. In this study, we analysed the predictive value of CTP in predicting outcomes of AVB patients. The results showed that CTP had the most excellent predictive power with the pooled AUC value of 0.824 in in-hospital mortality. The pooled sensitivity was highest (0.910, 95% CI: 0.864–0.944) with a specificity of 0.666 (95% CI: 0.635–0.696), which means CTP was superior to other risk scores in identifying patients who were at high risk of death in hospital. The predictive power was slightly declined in follow-up mortality with the pooled AUC value of 0.746. With a high pooled specificity of 0.806 and sensitivity of 0.722, CTP was also effective at triaging low-risk patients for early release or less intensive treatment, which had significant healthcare implications.

## MELD

MELD was first proposed by Malinchoc and later modified and improved by Malinchoc et al. and Kamath et al. [11,12]. The MELD score, according to Forman, was a valuable addition to the repertoire of prognostic instruments, and it seemed likely to dethrone the Child–Turcotte–Pugh method from its throne in the prognosis of chronic liver disease [45]. In contrast, Cholongitas stated that MELD did not perform better than the Child–Turcotte–Pugh score in non-transplant settings [46]. In this study, MELD had a lower pooled AUC value than CTP (AUC: 0.788 *vs.*0.824) in in-hospital mortality but had the highest pooled AUC value of 0.798 in follow-up mortality. That meant MELD was not as good as CTP in predicting in-hospital mortality but performed better in predicting outpatients outcomes in 3-month follow-up.

## GBS

Stanley suggested that the GBS could identify UGIB patients who can be managed safely as outpatients with an area under ROC curve of 0.90 [47]. An international multicentre prospective study involving 3012 patients showed that GSB was best (AUC: 0.86) at predicting intervention or death [48]. In that study, the number of patients with AVB was only 143 and accounted just for 7% among patients who had gone through endoscopy. In our meta-analysis, the results were different when there were only AVB patients. GBS showed low predictive power neither in mortality nor rebleeding outcomes with no AUC value more than 0.7. The cause might be that the GBS was developed in most ANVUGIB, which usually had a milder condition and better prognosis.

## AIMS65

The AIMS65 is a scoring system designed by Saltzman et al. on 29,222 patients’ clinical data analysis and integration. It is mainly used to assess the fatality rate of UGIB patients [9]. Hyett et al. compared AIMS65 and GBS in 278 UGIB patients and suggested that the AIMS65 score was superior in predicting inpatient mortality (AUC, 0.93 *vs.* 0.68, *p* < .001) [49]. The results in this meta-analysis concerning only AVB patients were similar to the previous research. AIMS65 performed better than GBS with a pooled AUC value of 0.793 in hospital and 0.77 in follow-up mortality.

### Rockall score

Rockall score was developed and established based on a prospective, unselected, multicentre study in 1996 [8]. Robertson reported that the full Rockall score had an AUC value of 0.78 in predicting AUGIB inpatient mortality based on 424 study patients [50]. Results were similar in AVB patients according to the results in our meta-analysis. The Full Rockall score had a good predictive power with a pooled AUC value of 0.75 in in-hospital mortality. However, it was low in follow-up mortality (AUC: 0.678). Not all patients had the chance to undergo endoscopy limited the application of full Rockall scores. To solve that problem, there came the clinical Rockall score. However, compared to other risk scores, the articles included concerning clinical Rockall score were decreased (*n* = 3), and the meta-analysis in in-hospital mortality was unable to conduct. The pooled AUC of follow-up mortality was 0.704, which showed that the clinical Rockall score had a moderate predictive power.

### Strengths and limitations

To our knowledge, no previous studies have conducted a meta-analysis to compare the predictive value of risk scores in AVB patients, despite that the AVB is life-threatening and patients would benefit most from risk stratification. Furthermore, the search was conducted in six different databases, allowing for greater comprehensiveness in the systematic search.

The small number of included studies of clinical and full Rockall scores when pooling sensitivity and specificity is a limitation in this meta-analysis.

When pooled sensitivity and specificity for the six risk scores, we found a high *I*^2^ statistic, indicating significant heterogeneity. The AVB patients included were most with cirrhosis. However, the aetiology of cirrhosis could be different, like virus hepatitis, alcohol and other reasons. What is more, some studies included AVB patients not only by cirrhosis but also other-cause portal hypertension. The different aetiology of oesophageal and gastric varices might cause selection bias. There was also clinical heterogeneity as studies used different follow-up time. We dealt with the clinical heterogeneity by performing subgroup analyses for different follow-up time. Artificial intelligence is showing considerable potential in risk stratification. Shung Dennis L developed and validated a machine learning odel for UGIB, showing 100% sensitivity with a specificity of 26% [51]. A similar method can be introduced in AVB patients, which could be more feasible and helpful.

### Addition to previous research

Ramaekers et al. to our knowledge, has performed a systematic study on the predictive value of risk scores in UGIB patients [52]. That study concluded all UGIB patients in the emergency department and did not perform subgroup analysis in AVB and ANVUGIB. Besides, the CTP and MELD score were not involved. The review concluded that GBS with a pooled sensitivity of 0.99 and a specificity of 0.08 (cutoff score = 0) was superior to other risk scores for identifying low-risk UGIB patients accurately. What is more, according to both US and UK guidelines, a GBS of zero was recommended to be used to classify very low-risk AUGIB patients who can avoid admission [4,6]. However, in our meta-analysis, the results were different. GBS showed low predictive power neither in mortality nor rebleeding with an AUC value no more than 0.7. Besides, with a pooled sensitivity of 0.783 and a specificity of 0.493 for overall mortality, GBS was not the best when compared to CTP with a pooled sensitivity of 0.848 and a specificity of 0.707. This indicated that GBS performed flawlessly, mainly in UGIB patients, especially in NVUGIB patients rather than AVB patients. However, only when the endoscopy has been completed would the causes of bleeding be known. The endoscopy is needed to achieve the highest accuracy resulting in GBS not being so favourable. Thus, if a patient is suspected of bleeding from varicose veins, it should be cautious when using GBS to identify low-risk patients. The CTP combined with GBS in risk stratification might be a safer but more complicated choice and needed further validation. Horibe M et al. developed a novel and simple scoring system, namely HARBINGER, to predict the outcomes for nonvariceal and variceal bleeding patients [53]. This study showed that the HARBINGER had greater accuracy than the GBS in predicting the urgency for an endoscopic intervention in all-cause UGIB patients (AUC, 0.74 *vs.* 0.63; *p* < .001). This simple score was further validated in a prospective multicentre Japanese setting involving 1486 patients. It showed that the new triage system set at one was proved accurate in ruling out the suspected UGIB patients with a sensitivity of 98.8% and specificity of 15.5% [54].

## Conclusion

No risk scores were ideally identified by our systematic review (CTP, MELD, GBS, AMIS65, full Rockall and clinical Rockall). CTP was superior over other risk scores in identifying AVB patients at high risk of death in hospital and patients at low risk of death within follow-up. Guidelines have recommended the use of GBS to risk stratification of patients with upper gastrointestinal bleeding. However, when it is suspected that the cause of upper gastrointestinal bleeding is from oesophageal and gastric varices, extra care should be taken. Because in this meta-analysis, it was found that the ability of GBS in predicting the death and rebleeding of AVB patients was limited. More researches are needed to validate it in the future. Artificial intelligence might be an important direction for future development to help risk-stratify AVB patients.

## Supplementary Material

Supplemental Material

## Data Availability

The study presents an analysis of data published in other studies.
