# Supplementary material for: Systematic review and meta-analysis of risk scores in prediction for the clinical outcomes in patients with acute variceal bleeding
Source: Ann Med. 2021 Oct 18;53(1):1806–15. doi: 10.1080/07853890.2021.1990394 (PMC8525940; doi:10.1080/07853890.2021.1990394)
Supplement: Supplemental Material [file IANN_A_1990394_SM2158.docx]

**Supplementary materials.**

**Prediction Scores:**

MELD = 0.957 × loge (creatinine mg/dL) +0.378 × loge (bilirubin mg/dL) +1.120 × loge (INR)+0.643 × (cause of cirrhosis). For cause of cirrhosis, use 0 for alcohol-related liver disease or for cholestatic liver disease; 1 for all other cause

Child-Pugh score parameters

| Parameters | 1 point | 2 points | 3 points |
| --- | --- | --- | --- |
| Serum bilirubin total (mg/dL) | < 34 (< 2) | 34-50 (2-3) | > 50 (> 3) |
| Serum albumin (mg/dL) | > 35 | 28–35 | < 28 |
| INR | < 1.7 | 1.71–2.20 | > 2.20 |
| Ascites | None | Suppressed with medication | Refractory |
| Hepatic encephalopathy | None | Grade I–II  (or suppressed with medication) | Grade III–IV  (or refractory) |

| Scoring system | Admission clinical factor | Parameter | Score |
| --- | --- | --- | --- |
| AIMS65 score | Albumin  INR  Mental status  SBP, mm Hg  Age, yr | <3.0 mg/dL  >1.5  Altered  ≤90  ≥65 | 1  1  1  1  1 |
| Glasgow-Blatchford score | BUN, mg/dL | ≥18.2 to <22.4  ≥22.4 to <28  ≥28 to <70  ≥70 | 2  3  4  6 |
|  | Hemoglobin level, g/dL | Male  ≥12.0 to <13.0  ≥10.0 to <12.0  <10.0  Female  ≥10.0 to <12.0  <10.0 | 1  3  6  1  6 |
|  | SBP, mm Hg | ≥100 to <109  ≥90 to <100  <90 | 1  2  3 |
|  | Other markers | Heart rate >100 bpm Presented with melena  Presented with syncope Hepatic disease  Cardiac failure | 1  1  2  2  2 |
| Rockall score | A:Age, yr | <60  60–79  ≥80 | 0  1  2 |
|  | B:Shock | Heart rate >100 bpm SBP <100 mm Hg | 1  2 |
|  | C:Comorbidity | No major  CHF, IHD, or major comorbidity  Renal failure, liver failure, metastatic malignancy | 0  2  3 |
|  | D:Endoscopic finding | Mallory–Weiss tear or no lesion and no stigmata  All other diagnoses  GI malignancy | 0  1  2 |
|  | E:Stigmata of recent bleeding | No stigmata or pigmented spot on ulcer  Blood in upper GI tract, adherent clot, visible vessel, bleeding | 0  2 |
|  |  | A+B+C=clinical rockall  A+B+C+D+E=full rockall score |  |

search strategy

**PUBMED**

((((((((("risk scor*"[Text Word]) OR ("scoring system*"[Text Word])) OR ("risk stratification*"[Text Word])) OR ("risk assessment*"[Text Word])) OR (scor*[Text Word])) OR ("Severity of Illness Index*"[Text Word])) OR ("Risk Assessment*"[Text Word])) OR ("Severity of Illness Index"[**MeSH Terms**])) OR ("Risk Assessment"[**MeSH Terms**])) AND ((((((((("Gastrointestinal Hemorrhage"**[Mesh]**) OR ("Gastrointestinal Hemorrhage"[TW])) OR ("Hemorrhage, Gastrointestinal"[TW])) OR ("Gastrointestinal Hemorrhages"[TW])) OR ("Hematochezia"[TW])) OR ("Hematochezias"[TW])) OR (((("Hemorrhage"**[Mesh])** OR ("Hemorrhage"[TW])) OR ("Hemorrhages"[TW])) OR ("Bleeding"[TW]))) AND (((((((((("Esophageal and Gastric Varices"**[Mesh]**) OR ("Esophageal and Gastric Varices"[Text Word])) OR ("Gastric Varix"[Text Word])) OR ("Varices, Gastric"[Text Word])) OR ("Gastric Varices"[Text Word])) OR ("Esophageal Varices"[Text Word])) OR ("Esophageal Varix"[Text Word])) OR ("Varices, Esophageal"[Text Word])) OR ("Varix, Esophageal"[Text Word])) OR ("gastroesophageal varices"[Text Word]))) OR ("variceal bleeding"[TW]))

**WEB of science**

**#1**TS= ("risk scor*" OR "scoring system*" OR "risk stratification*" OR "risk assessment*" OR scor* OR "Severity of Illness Index*" OR "Risk Assessment*")

**#2**TS= ("Esophageal and Gastric Varices" OR "Gastric Varix" OR "Varices, Gastric" OR "Varix, Gastric" OR "Gastric Varices" OR "Esophageal Varices" OR "Esophageal Varix" OR "Varices, Esophageal" OR "Varix, Esophageal" OR "gastroesophageal varices"）

**# 3**TS=("Hemorrhage*" OR "Bleeding")

**#4**TS=("Gastrointestinal Hemorrhage" OR "Hemorrhage, Gastrointestinal" OR "Gastrointestinal Hemorrhages" OR "Hematochezia" OR "Hematochezias")

**# 5**TS=("variceal bleeding")

**# 6**#4 OR #3

**# 7**#6 AND #2

**# 8**#7 OR #5

**# 9**#8 AND #1

**COCHRANE**

**#1** ("risk score"):ti,ab,kw OR ("scoring system"):ti,ab,kw OR ("risk stratification"):ti,ab,kw OR (“risk assessment”):ti,ab,kw OR (score):ti,ab,kw (Word variations have been searched)

**#2** ("Severity of Illness Index"):ti,ab,kw

**#3**MeSH descriptor: [Risk Assessment] explode all trees

**#4**MeSH descriptor: [Severity of Illness Index] explode all trees

**#5** #1 or #2 or #3 or #4

**#6** ("Esophageal and Gastric Varices"):ti,ab,kw OR ("Gastric Varix"):ti,ab,kw OR ("Varices, Gastric"):ti,ab,kw OR ("Gastric Varices"):ti,ab,kw OR ("Esophageal

**#7** ("Esophageal Varix"):ti,ab,kw OR ("Varices, Esophageal"):ti,ab,kw OR ("Varix, Esophageal"):ti,ab,kw OR ("gastroesophageal varices"):ti,ab,kw

**#8** #6 or #7（2507）

**#9** MeSH descriptor: [Esophageal and Gastric Varices] explode all trees

**#10** #8 or #9

**#11** ("Gastrointestinal Hemorrhage"):ti,ab,kw OR ("Hemorrhage, Gastrointestinal"):ti,ab,kw OR ("Gastrointestinal Hemorrhages"):ti,ab,kw OR ("Hematochezia"):ti,ab,kw OR (Hemorrhage):ti,ab,kw

**#12** (Bleeding):ti,ab,kw

**#13** MeSH descriptor: [Hemorrhage] explode all trees

**#14** MeSH descriptor: [Gastrointestinal Hemorrhage] explode all trees

**#15** #11 or #12 or #13 or #14

**#16** #10 and # 15

**#17** ("variceal bleeding"):ti,ab,kw

**#18** #16 or #17

**#19** #5 and #18

**EMBASE**

**#1** ((('esophagus varices'/exp OR ('esophageal and gastric varices' OR 'gastric varix' OR 'varices, gastric' OR 'varix, gastric' OR 'gastric varices' OR 'esophageal varices' OR 'esophageal varix' OR 'varices, esophageal' OR 'varix, esophageal' OR 'gastroesophageal varices')) AND ('bleeding'/exp OR ('hemorrhage' OR 'hemorrhages' OR 'bleeding') OR

'gastrointestinal hemorrhage'/exp OR ('gastrointestinal hemorrhage' OR 'hemorrhage, gastrointestinal' OR 'gastrointestinal hemorrhages' OR 'hematochezia' OR 'hematochezias'))) OR ('variceal bleeding' OR 'esophagus varices bleeding'/exp))

**#2**'risk scor*':ti,ab,kw OR 'scoring system*':ti,ab,kw OR 'risk stratification*':ti,ab,kw OR scor*:ti,ab,kw OR 'severity of illness index*':ti,ab,kw OR 'risk assessment*':ti,ab,kw

**#3** #1 and #2

**CNKI：**“variceal bleeding" and "risk scor*"

**WANFANG：**“variceal bleeding" and "risk scor*"
